# Central Pathology Review in SENTIX, a Prospective Observational International Study on Sentinel Lymph Node Biopsy in Patients with Early-Stage Cervical Cancer (ENGOT-CX2)

**DOI:** 10.3390/cancers12051115

**Published:** 2020-04-29

**Authors:** Kristyna Nemejcova, Roman Kocian, Christhardt Kohler, Jiri Jarkovsky, Jaroslav Klat, Alberto Berjon, Radovan Pilka, Borek Sehnal, Blanca Gil-Ibanez, Ezequiel Lupo, Almerinda Petiz, Octavio Arencibia Sanchez, Peter Kascak, Fabio Martinelli, Alessandro Buda, Jiri Presl, Marc Barahona, Luc van Lonkhuijzen, Wiktor Szatkowski, Lubos Minar, Maja Pakiz, Pavel Havelka, Cristina Zorrero, Marcin Misiek, Leon Cornelius Snyman, Dariusz Wydra, Ignace Vergote, Alla Vinnytska, Mikulas Redecha, Martin Michal, Solveig Tingulstad, Barbara Kipp, Grzegorz Szewczyk, Robert Toth, Francisco Javier de Santiago Garcia, Pluvio Jesus Coronado Martin, Robert Poka, Karl Tamussino, Mathieu Luyckx, Maxime Fastrez, Juan Carlos Staringer, Anna Germanova, Andrea Plaikner, Sylva Bajsova, Pavel Dundr, Nina Mallmann-Gottschalk, David Cibula

**Affiliations:** 1 Institute of Pathology, First Faculty of Medicine, Charles University and General University Hospital, 12000 Prague, Czech Republic; kristyna.nemejcova@vfn.cz (K.N.); pavel.dundr@vfn.cz (P.D.); 2 Gynecologic Oncology Center, Department of Obstetrics and Gynecology, First Faculty of Medicine, Charles University and General University Hospital, 12000 Prague, Czech Republic; roman.kocian@vfn.cz (R.K.); ena.german@gmail.com (A.G.); 3 Department of Special Operative and Oncologic Gynaecology, Asklepios-Clinic Hamburg, 22763 Hamburg, Germany; ch.koehler@asklepios.com (C.K.); an.plaikner@gmail.com (A.P.); 4 Institute for Biostatistics and Analyses, Faculty of Medicine, Masaryk University, 62500 Brno, Czech Republic; jarkovsky@iba.muni.cz; 5 Department of Obstetrics and Gynecology, University Hospital Ostrava, 708 52 Ostrava Poruba, Czech Republic; jaroslav.klat@fno.cz (J.K.); sylva.bajsova@fno.cz (S.B.); 6 Department of Pathology, La Paz University Hospital, 28046 Madrid, Spain; alberto.berjon@gmail.com; 7 Molecular Pathology and Therapeutic Targets Group, IdiPAZ, 28046 Madrid, Spain; 8 Department of Obstetrics and Gynecology, Faculty of Medicine and Dentistry, Palacky University, University Hospital Olomouc, 77520 Olomouc, Czech Republic; radovan.pilka@fnol.cz; 9 Department of Obstetrics and Gynecology, Bulovka Hospital, First Faculty of Medicine, Charles University, 18081 Prague, Czech Republic; boreksehnal@seznam.cz; 10 Unit of Gynecological Oncology, Institute Clinic of Gynecology, Obstetrics and Neonatology (ICGON), Hospital Clinic of Barcelona, 08036 Barcelona, Spain; BLGIL@clinic.cat; 11 Department of Pathology, Institute of Oncology Angel H. Roffo, University of Buenos Aires, Buenos Aires Autonomous City 1214, Argentina; negrolupo@yahoo.com.ar; 12 Department of Gynecology, Francisco Gentil Portuguese Oncology Institute, 4200-072 Porto, Portugal; almerindapetiz@hotmail.com; 13 Departments of Gynecologic Oncology, University Hospital of the Canary Islands, 35016 Las Palmas de Gran Canaria, Spain; octavaren@hotmail.com; 14 Department of Obstetrics and Gynecology, Faculty Hospital Trencin, 91171 Trencin, Slovakia; pkascak@gmail.com; 15 IRCCS Foundation National Cancer Institute in Milan, 20133 Milan, Italy; fabio.martinelli@istitutotumori.mi.it; 16 Department of Obstetrics and Gynecology, Unit of Gynecologic Oncology Surgery, San Gerardo Hospital, 20900 Monza, Italy; alebuda1972@gmail.com; 17 Department of Gynaecology and Obstetrics, University Hospital Pilsen, Charles University, 30460 Prague, Czech Republic; jiri.presl77@gmail.com; 18 Department of Gynecology, University Hospital of Bellvitge, Biomedical Research Institute of Bellvitge, University of Barcelona, 08907 Barcelona, Spain; mbarahona@bellvitgehospital.cat; 19 Center for Gynecologic Oncology, Academic Medical Centre, 1100DD Amsterdam, The Netherlands; l.r.vanlonkhuijzen@amc.uva.nl; 20 Department of Gynecologic Oncology, Centre of Oncology, M. Sklodowska-Curie Memorial Institute, Cracow Department, 31-115 Cracow, Poland; vigor27@wp.pl; 21 Department of Gynecology and Obstetrics, Faculty of Medicine, Masaryk University, 60200 Brno, Czech Republic; minar.lubos@fnbrno.cz; 22 University Clinic for Gynaecology and Perinatology, University Medical Centre Maribor, 2000 Maribor, Slovenia; maja.pakiz@ukc-mb.si; 23 KNTB a.s, 762 75 Zlin, Czech Republic; havelka@bnzlin.cz; 24 Gynecology Department, Instituto Valenciano de Oncología (IVO), 46009 Valencia, Spain; czorrero@gmail.com; 25 Department of Gynecologic Oncology, Holycross Cancer Center, 25-734 Kielce, Poland; marcin.misiek@me.com; 26 Gynaecologic Oncology Unit, Department of Obstetrics and Gynaecology, University of Pretoria, Pretoria 0001, South Africa; leon.snyman@up.ac.za; 27 Department of Gynecology, Gynecologic Oncology and Gynecologic Endocrinology, Medical University of Gdansk, 80-402 Gdansk, Poland; dwydra@uck.gda.pl; 28 Department of Gynecology and Obstetrics, University Hospital Leuven, Leuven Cancer Institute, 3000 Leuven, Belgium; ignace.vergote@uzleuven.be; 29 LISOD-Israeli Oncological Hospital, 08720 Plyuty, Ukraine; alla.vinnytska@lisod.ua; 30 Department of Gynaecology and Obstetrics, University Hospital, Comenius University, 82101 Bratislava, Slovakia; mikulasredecha@gmail.com; 31 Department of Obstetrics and Gynaecology, Hospital Ceske Budejovice, JSC, 37001 Ceske Budejovice, Czech Republic; martinmichal@email.cz; 32 Department of Gynaecology, St Olav’s Hospital, 7006 Trondheim, Norway; solveig.tingulstad@stolav.no; 33 Department of Obstetrics and Gynecology, Cantonal Hospital of Lucerne, 6000 Lucerne, Switzerland; barbara.kipp@luks.ch; 34 Department of Obstetrics and Gynecology, Institute of Mother and Child, 01-211 Warsaw, Poland; grzegorz.szewczyk@wum.edu.pl; 35 Oncology Institute of East Slovakia, 04191 Košice, Slovakia; toth@vou.sk; 36 MD Anderson Cancer Center, 28033 Madrid, Spain; fdesantiago@mdanderson.es; 37 Department of Gynecology and Obstetrics, Hospital Clinico San Carlos, 28040 Madrid, Spain; pcoronadom@gmail.com; 38 Institute of Obstetrics and Gynaecology, Faculty of Medicine, University of Debrecen, 4032 Debrecen, Hungary; pokar@med.unideb.hu; 39 Medical University of Graz, Graz 8036, Austria; karl.tamussino@medunigraz.at; 40 Department of Gynecology, Universite catholique de Louvain, Cliniques Universitaires St Luc, 1200 Brussels, Belgium; mathieu.luyckx@uclouvain.be; 41 Department of Obstetrics and Gynaecology, St Pierre University Hospital, Universite Libre de Bruxelles, 1000 Brussels, Belgium; maxime_fastrez@stpierre-bru.be; 42 Department of Gynecology and Obstetrics, Hospital Español de Buenos Aires, Buenos Aires 2975, Argentina; jstaringer@hotmail.com; 43 Department of Gynecology, Medical Faculty, University of Cologne, 50937 Cologne, Germany; nina.gottschalk@web.de

**Keywords:** sentinel lymph node, cervical cancer, metastases

## Abstract

The quality of pathological assessment is crucial for the safety of patients with cervical cancer if pelvic lymph node dissection is to be replaced by sentinel lymph node (SLN) biopsy. Central pathology review of SLN pathological ultrastaging was conducted in the prospective SENTIX/European Network of Gynaecological Oncological Trial (ENGOT)-CX2 study. All specimens from at least two patients per site were submitted for the central review. For cases with major or critical deviations, the sites were requested to submit all samples from all additional patients for second-round assessment. From the group of 300 patients, samples from 83 cases from 37 sites were reviewed in the first round. Minor, major, critical, and no deviations were identified in 28%, 19%, 14%, and 39% of cases, respectively. Samples from 26 patients were submitted for the second-round review, with only two major deviations found. In conclusion, a high rate of major or critical deviations was identified in the first round of the central pathology review (28% of samples). This reflects a substantial heterogeneity in current practice, despite trial protocol requirements. The importance of the central review conducted prospectively at the early phase of the trial is demonstrated by a substantial improvement of SLN ultrastaging quality in the second-round review.

## 1. Introduction

SENTIX is an observational prospective study that was designed to evaluate whether a less radical surgical approach comprising sentinel lymph node (SLN) biopsy could replace systematic pelvic lymphadenectomy (PLND) in the management of patients with early-stage cervical cancer [1]. The primary endpoint of the study is the recurrence rate at 24 months after surgery.

Metastatic involvement of pelvic lymph nodes (LN) is the most important prognostic marker in patients with early-stage cervical cancer [2,3,4,5,6,7,8,9,10,11]. Since non-SLN pelvic LNs are not surgically removed in the study, each case with undetected LN involvement can cause a lateral pelvic recurrence, which is often fatal for the patient. SLN pathological ultrastaging improves the sensitivity of LN staging thanks to the more reliable detection of small metastases that would otherwise be missed by standard evaluation [8,9,12,13,14,15,16]. The quality of the SLN pathological evaluation is considered the key prerequisite for the safety of patients in the study. Therefore, the central pathology review was an integral part of the protocol. Samples from two randomly selected patients were submitted for central review from each institution. If the outcome of the review was classified as having major or critical deviations, which could result in missing metastatic involvement, samples from all cases from that institution were requested for the second round of the review.

Standard pelvic lymph node dissection is not performed in the SENTIX trial. Instead, SLNs are removed on both sides of the pelvis, and the major lymphatic channels draining lymph from the lower extremities are preserved. The main advantage of this less radical approach is the substantially lower risk of postoperative complications, such as lower leg lymphedema and pelvic lymphoceles [17,18,19,20]. Lowering the number of removed LNs should, however, be compensated by their more intensive pathology assessment. Protocols for SLN ultrastaging were developed to detect smaller metastases and improve the reliability of SLN staging [12,14,21,22,23]. In the largest retrospective cohort study on sensitivity of SLN ultrastaging, 645 patients underwent SLN biopsy followed by pelvic lymph node dissection [24]. In 23 (3.6%) patients, pathological ultrastaging detected MIC in their SLN, while larger macrometastases were identified in the other pelvic LN. These patients would not be identified as high-risk patients if surgical staging was limited only to SLN biopsy and SLN ultrastaging was not performed. Neither of their pelvic LNs would be removed, nor would they have received adjuvant radiotherapy, with a subsequent significant risk of developing lateral pelvic recurrence with a low chance of curative treatment.

To our knowledge, SENTIX is the first prospective study in patients with cervical cancer that included a prospective review of specimens from SLN. In this paper, we report the final results of the Central Pathology Review for the 300 patients treated per protocol.

## 2. Results

A total of 47 sites across 18 countries were registered to participate in the SENTIX study. To the date when the first 300 cases were registered and treated according to the protocol, 37 sites enrolled at least two cases, two sites enrolled one case, and eight sites enrolled no patients. The characteristics of the group are shown in Table 1. The outcome of the central review was concluded eight months after the date the 300th patient was enrolled, when all requested samples were received and reviewed.

Thirty-seven sites were eligible to submit samples for the first-round review. Samples from 83 patients treated in 35 sites were reviewed, including three cases from the trial leading institution. Samples from two Argentinian sites were missing because of customs and transportation challenges. Original pathology reports from two Argentinian sites were, nevertheless, translated into English, and the protocol of SLN assessment reviewed.

A central pathology review classified findings from the first round as having no deviations in 32 (39%) cases, minor deviations in 23 (28%), major deviations in 16 (19%), and critical in 12 (14%) cases. This corresponds to eight and six sites, respectively, with at least one case with major or critical deviations. SLNs were not processed completely in 40% of cases, and immunohistochemical staining was performed less frequently than required by the protocol in 25% of cases and not at all in 11% of cases. Surprisingly, there were two cases with a higher number of immunohistochemical staining. Other minor issues were found in 16% of cases. These included the use of a different staining sequence or using different immunohistochemical/histochemical staining (i.e., cytokeratin-7 with periodic acid–Schiff or Papanicolaou staining).

**Figure 1:**
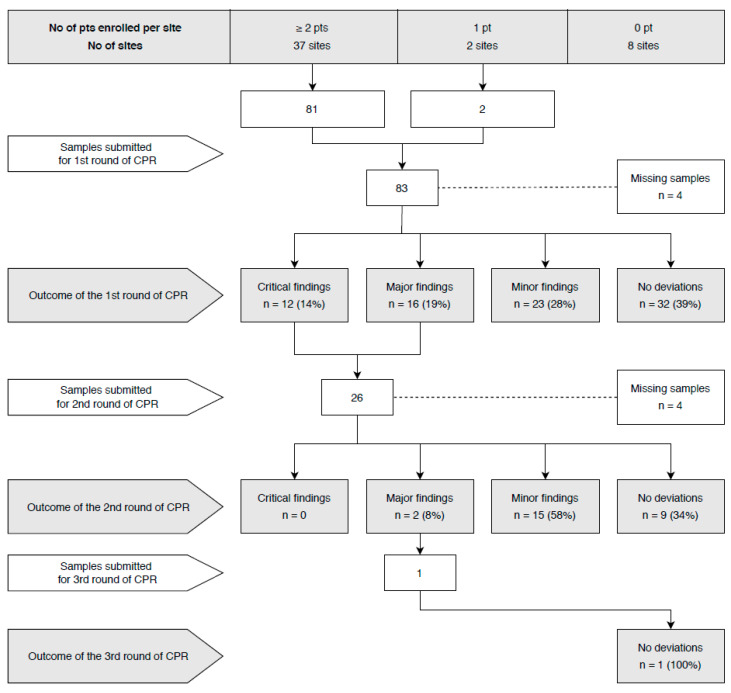
Flow chart of the central pathology review (CPR).

For the second-round review, nine sites with major or critical deviations in the first round were asked to submit samples from all enrolled cases. Four sites had not enrolled any other patients at the time of the review, and two centers were prematurely closed. In 26 submitted cases for the second-round review, no deviations were found in nine (35%), while minor deviations were found in 15 (58%), and major deviations in two (8%) cases. One site with major deviations detected in the first and second rounds submitted samples from patients enrolled later in the study for the third-round review, resulting in no deviation. Figure 1 shows the flow chart of the central pathology review. Two sites were prematurely terminated due to critical deviations in the first round, poor communication, and no attempt to resolve the identified issues after repeated requests. Patients from these sites were excluded from the per-protocol analysis.

From the whole cohort of 300 patients, samples from 110 cases (37%) were reviewed in the central laboratory (83 in the first, 26 in the second, and 1 in the third round). Samples from 350 SLNs consisting of 262 in the first round, 85 in the second round, and 3 in the third round were reviewed. Eight micrometastases and two ITCs were found by the initial pathology evaluation at referring institutions. Cases with residual SLN tissue in paraffin blocks, which constituted major or critical deviations, were reprocessed according to the study protocol at the central laboratory, yielding 1782 additional slides. Two additional micrometastases were found in two cases.

## 3. Discussion

The central review of SLN pathological assessment was an integral part of the ongoing prospective observational SENTIX trial. The main objective of the study was to evaluate the safety of bilateral SLN biopsy instead of systematic pelvic lymph node dissection in patients with early-stage cervical cancer. According to the protocol, the outcome of central pathology review was analyzed in the group of 300 patients treated per protocol. Samples from 110 cases from 37 sites were submitted to the central laboratory. The central review revealed a high number of deviations from the trial protocol for SLN pathological ultrastaging. In the first round, major or critical deviations, defined as those which could potentially result in missing metastatic involvement, were found in 34% of reviewed cases. The most frequent type of deviation was residual tissue found in the paraffin block due to incomplete processing of SLN tissue.

Central review has been conducted continuously throughout the period spanning January 2017 to May 2019. All deviations from the protocol have been reported to the referring sites and communicated to all investigators. We believe that this quality control process led to substantial improvement in pathological SLN ultrastaging quality, demonstrated by the results of the second and third rounds of the review, where only two (8%) major and zero critical deviations were reported. Two sites were terminated due to insufficient quality of pathology examination, and patients from these sites were excluded from the per-protocol analysis.

We are aware that the quality of pathological SLN evaluation is the critical element for the study outcome and safety of patients. One of the main conditions for site selection demanded experience with SLN biopsy in gynecological malignancies and experience with SLN ultrastaging. Institutional pathologists had to agree with the protocol for SLN ultrastaging and with the central review. Therefore, the high frequency of serious deviations reported in the first round of the review was an unexpected revelation. We can hypothesize that the underlying cause is insufficient communication between clinicians and their pathologists. This outcome also reflects substantial differences in protocols for SLN evaluation between institutions in the absence of internationally accepted recommendations. In a recently published review article, we showed that reported protocols for SLN ultrastaging differ substantially; they are often incompletely described or they are not reported at all [25].

The prevalence of micrometastases varies around 10% in patients with early-stage cervical cancer [24,26]. It approximately corresponds to the results of our study where 10 (9%) cases with micrometastases were found. In 132 SLNs from 40 cases that were reprocessed at the central laboratory, we detected only two additional cases with MIC that were missed at a referring institution. A plausible explanation for the slightly lower than expected occurrence of MIC is preselection of patients. According to the protocol, MRI or expert ultrasound was mandatory in preoperative staging, and all cases with enlarged or suspicious pelvic LN were excluded from the trial. Cases with intraoperatively detected LN involvement were also excluded.

One of the limitations of our protocol for central review in the trial was the timing of central review that started early after the study initiation. Thus, quality control was conducted earlier in sites with faster recruitment. Another weakness is the fact that samples were not reviewed from all enrolled cases. We believe, however, that the algorithm for patient selection was a reasonable compromise which allowed for prospective quality control in the early phase of the trial and improvement of the quality of pathological assessment in the trial.

We would like to emphasize the strong features of the central pathology review design. In the context of other published protocols in cervical cancer, ultrastaging was very intense, with the intention to guarantee reliability in the detection of all metastases ≥200 µm, including small MAC and MIC. The review of samples was performed continuously during the first study period until the first 300 patients were enrolled. If the review had been conducted retrospectively after all patients were registered, the logistics would have been much easier; however, it would not have allowed the real-time communication of findings with sites and quality improvement. Two rounds of the review allowed us to focus attention on sites with difficulties in pathological assessment. The outcome of the second round confirmed a substantial performance improvement. We have developed a meticulous reporting system of review outcomes and a grading of findings. In cases of major deviations, SLN assessment was completed according to the protocol at the central laboratory, without the need of additional sample transportation back to the referring sites. Patients scheduled for fertility-sparing surgery were eligible in the trial since they could profit from intraoperative triage the most [27,28,29]. The threshold for upper tumor size in fertility-sparing treatment was 2 cm, which corresponds to the recent international recommendations [9].

## 4. Materials and Methods

### 4.1. Ethics

The protocol was approved by the IRB of the leading institution (General University Hospital in Prague, project number/ethic code: 105/15 IRB of the General Faculty Hospital in Prague) in June 2016, and institutional IRB approval has been a prerequisite for participation for each new institution. Information for patients was available in 17 languages, and informed consent was signed by all patients before preregistration into the study. Study registration: NCT02494063 (ClinicalTrials.gov); European Network of Gynaecological Oncological Trial (ENGOT)-CX2; Central and Eastern European Gynecologic Oncology Group (CEEGOG)-CX1.

### 4.2. Study Sites

Sites applied individually for participation in the study, and eligibility criteria were evaluated by the SENTIX study office. The minimum requirements for any site to participate included a minimum of 10 patients with early-stage invasive cervical carcinoma treated in the center per year, experience with at least 15 gynecological cancer patients with successful SLN detection, and approval of the protocol for pathological SLN ultrastaging by pathologists.

The SENTIX study was conducted in collaboration with ENGOT (European Network of Gynaecological Oncological Trial groups), according to ENGOT Model A [30].

### 4.3. Patients

**Figure 2:**
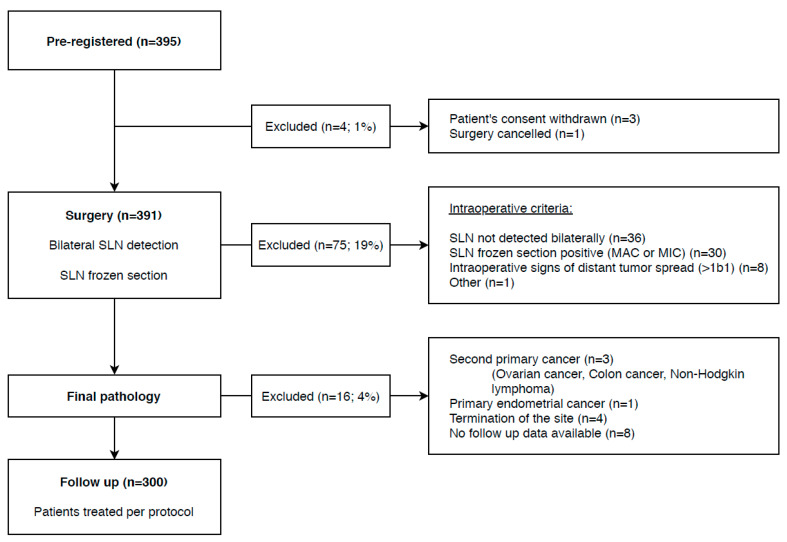
Flow chart for patients registered into the SENTIX trial.

Patients were preregistered into the study if they fulfilled the following inclusion criteria: (a) FIGO classification 2014 [31] stage IA1 + lymphovascular space invasion (LVSI), IA2, IB1; (b) pelvic LNs not enlarged or suspicious on preoperative imaging; (c) squamous cell carcinoma, adenocarcinoma, or adenosquamous carcinoma; (d) tumor with the largest diameter of ≤4 cm or ≤2 cm in patients scheduled for a fertility-sparing procedure. Patients after neoadjuvant chemotherapy or those with an unusual type of adenocarcinoma (non-HPV-related, e.g., mucinous, clear cell, mesonephric) were excluded. Final registration was provided after the surgery if additional intraoperative criteria were met, such as successful bilateral SLN detection, negative SLN frozen section evaluation, and no intraoperative evidence of more advanced disease (>IB1). The flow chart for patients is displayed in Figure 2.

### 4.4. SLN Detection

All surgical approaches and all 3 main, currently available techniques for SLN detection, such as blue dye, radiocolloid, or indocyanine green, were eligible by the protocol. However, preferred techniques were either a combination of blue dye and radiocolloid or indocyanine green [10,14,32,33,34].

### 4.5. SLN Ultrastaging Protocol

**Figure 3:**
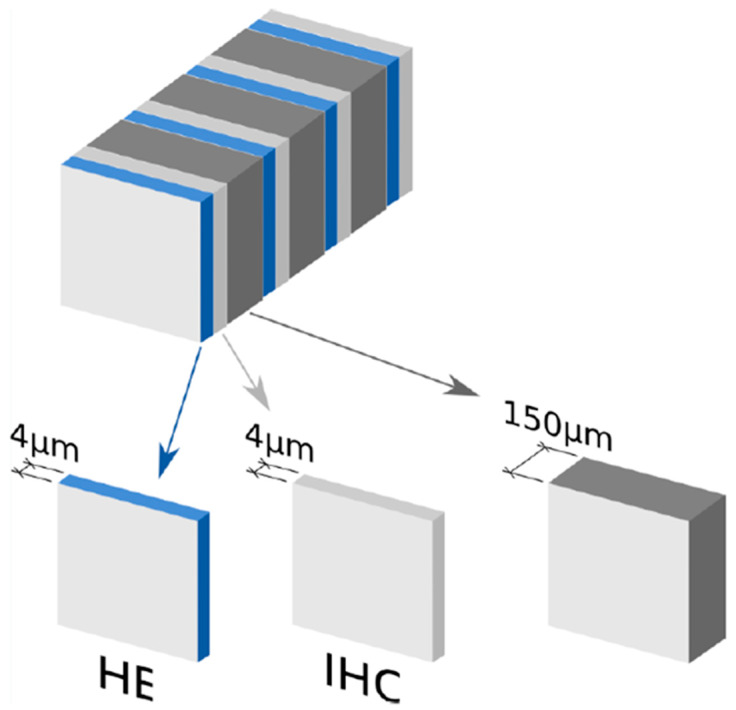
Sentinel lymph node (SLN) ultrastaging protocol in SENTIX trial protocol.

After intraoperative processing, all SLNs were fixed in 10% neutral buffered formalin, sliced at 2 mm intervals, and embedded in paraffin. The tissue sections were then processed for ultrastaging. Pairs of tissue sections (4 μm thick) were cut at 150 μm intervals in a serial manner from each paraffin block until there was no lymph node tissue left. The first section of each pair was stained with hematoxylin and eosin (HE), and the second section was examined immunohistochemically after staining with anticytokeratin AE1/AE3 antibodies (Figure 3). The protocol was mandatory for all participating institutions.

The type of metastasis was classified according to the TNM system [35]. Macrometastases were defined as metastases >2 mm in diameter, micrometastases as metastasis of >0.2 to ≤2 mm in diameter, and ITCs as individual cells or small clusters of cells up to 0.2 mm in diameter (<200 cells).

### 4.6. Central Pathology Review

According to the protocol, all SLN slides with corresponding paraffin blocks and the full pathology report from 2 patients per site were requested by the trial coordinator for central review. Cases were selected randomly from patients without MAC, reported by referring sites using a random number generator. The trial coordinator was responsible for communication with sites, random selection of cases, logistics of sample transportation, and review report finalization and submission to the sites. Samples were reviewed by the central laboratory at the Department of Pathology of the SENTIX leading institution at the General University Hospital in Prague, Czech Republic. All samples were reviewed by one of the two senior gynecological pathologists. The review process included examination of all slides, review of the original pathology report, correlation of the number of slides with a size and number of lymph nodes, checking the number of immunohistochemical slides, and examination of paraffin blocks for any residual unprocessed lymph node tissue.

The outcome of each review was summarized in a report composed of: (a) description of received samples and original findings at the referring institution, (b) description of deviations from the protocol, (c) grading of deviations, (d) description and findings of SLN evaluation completed according to the protocol at the central laboratory (in cases with major or critical deviation), (e) final SLN status combining results from SLN evaluation at the referring institution and at the central laboratory, (f) recommendation for quality improvement if any deviations were identified. The report also contained a copy of SLN protocol from the SENTIX trial and a description of the grading system of deviations.

The outcome of the central review was graded as follows: (1) No deviations, if all SLNs were processed according to the study protocol. (2) Minor deviations, if samples were processed with high quality with minor deviations carrying no risk to miss metastatic involvement (MAC or MIC), i.e., a lower number of IHC staining. (3) Major deviations, if deviations were found which could result in failure to detect metastasis and if the assessment could be completed by additional assessment at the central laboratory (i.e., SLN processed incompletely or no IHC staining). (4) Critical deviations, if major deviations were found but the assessment could not be completed in the central laboratory due to incomplete sample submission. In cases with major deviations, residual SLN tissues in paraffin blocks were reprocessed according to the study protocol at the central laboratory. If critical deviations were identified, sites were requested to complete SLN evaluation according to the protocol and return these samples for the second or, if necessary, the third review round. All major and critical deviations were discussed with the site investigator and pathologist.

All sites with major or critical deviations were asked to submit samples and pathology reports from all enrolled patients for the second round of assessment. The same grading classification and reporting were applied to the second-round assessment. A summary of the outcomes and recommendations for the improvement of SLN assessment was also reported in quarterly newsletters distributed to all study investigators.

According to the original protocol, the central pathology review was planned in the first 300 cases registered into the trial and treated by the protocol. This milestone was achieved on 27 September 2018. By that date, 395 patients were preregistered. The patient flow chart is shown in Figure 2.

## 5. Conclusions

The high number of severe deviations from the SLN ultrastaging protocol was an unexpected outcome of the first round of the central pathology review in the SENTIX trial. The central review and an intensive communication of its outcome with investigators resulted in a substantial improvement in the quality of pathological assessments, as demonstrated by the outcomes of the second-round review. The results of this study highlight the need for a central pathology review in similar studies involving multiple sites in multiple countries, where different protocols may otherwise be used in routine clinical practice.

## Figures and Tables

**Table 1:** Preoperative characteristics of patients (*N* = 300).

Characteristics	Values	*N* (%)
Site category according to number of enrolled patients	≤10	150 (50%)
	11–20	39 (13%)
	>20	111 (37%)
Age (continuous)		41 (29; 65)
Age category	≤40	129 (43%)
	41–60	139 (46.3%)
	>60	32 (10.7%)
BMI	≤25	172 (57.3%)
	26–30	68 (22.7%)
	>30	59 (19.7%)
	Missing	1 (0.3%)
ECOG performance status	0	287 (95.7%)
	1	12 (4.0%)
	Missing	1 (0.3%)
No. of prior pregnancies	0	64 (21.3%)
	1	53 (17.7%)
	2	99 (33%)
	>2	83 (27.7%)
	Missing	1 (0.3%)
No. of prior deliveries	0	77 (25.7%)
	1	74 (24.7%)
	2	102 (34%)
	>2	46 (15.3%)
	Missing	1 (0.3%)
Diagnostic procedure Biopsy		118 (39.3%)
Conization		185 (61.7%)
Stage (preoperative)	T1a1 + LVSI	16 (5.3%)
	T1a2	24 (8.0%)
	T1b1	259 (86.3%)
	Missing	1 (0.3%)
Grade	G1	72 (24.0%)
	G2	160 (53.3%)
	G3	64 (21.3%)
	Missing	4 (1.3%)
Tumor type	Squamous cell carcinoma	211 (70.3%)
	Adenocarcinoma usual type	84 (28.0%)
	Adenosquamous carcinoma	4 (1.3%)
	Missing	1 (0.3%)
Tumor size	≤2 cm	209 (69.7%)
	>2 cm	90 (30.0%)
	Missing	1 (0.3%)
LVSI	Yes	86 (28.7%)
	No	210 (70.0%)
	Missing	4 (1.0%)
Number of SLN	2	127 (42.3%)
	3	86 (28.7%)
	4	45 (15.0%)
	>4	42 (13.9%)
Fertility-sparing surgery (FSS)	All FSS Conization Simple trachelectomy Radical trachelectomy	52 (17.3%) 66675 (1.7%) 666719 (6.3%) 666728 (9.3%)
